# Revisiting Steroidogenic Pathways in the Human Placenta and Primary Human Trophoblast Cells

**DOI:** 10.3390/ijms22041704

**Published:** 2021-02-08

**Authors:** Rona Karahoda, Sampada Kallol, Michael Groessl, Edgar Ontsouka, Pascale Anderle, Christa Fluck, Frantisek Staud, Christiane Albrecht

**Affiliations:** 1 Department of Pharmacology and Toxicology, Faculty of Pharmacy in Hradec Kralove, Charles University, Akademika Heyrovskeho 1203, 500 05 Hradec Kralove, Czech Republic; karahodr@faf.cuni.cz; 2 Institute of Biochemistry and Molecular Medicine, University of Bern, Bühlstrasse 28, 3012 Bern, Switzerland; sampada.kallol@ibmm.unibe.ch (S.K.); edgar.ontsouka@ibmm.unibe.ch (E.O.); 3 Swiss National Centre of Competence in Research (NCCR) TransCure, University of Bern, 3012 Bern, Switzerland; 4 Department of Nephrology and Hypertension, Department of Biomedical Research, Inselspital, University of Bern, Freiburgstrasse 15, 3010 Bern, Switzerland; michael.groessl@dbmr.unibe.ch; 5 Sitem Center for Translational Medicine and Biomedical Entrepreneurship and Sitem-Insel AG, University of Bern, Freiburgstrasse 3, 3010 Bern, Switzerland; pascale.anderle@sitem.unibe.ch; 6 Pediatric Endocrinology, Diabetology and Metabolism, Department of Pediatrics, Department of Biomedical Research, Inselspital, Bern University Hospital, University of Bern, Freiburgstrasse 15, 3010 Bern, Switzerland; christa.flueck@dbmr.unibe.ch

**Keywords:** steroidogenesis, steroid metabolism, placenta, gestation, trophoblast

## Abstract

Steroid hormones play a crucial role in supporting a successful pregnancy and ensuring proper fetal development. The placenta is one of the principal tissues in steroid production and metabolism, expressing a vast range of steroidogenic enzymes. Nevertheless, a comprehensive characterization of steroidogenic pathways in the human placenta and potential developmental changes occurring during gestation are poorly understood. Furthermore, the specific contribution of trophoblast cells in steroid release is largely unknown. Thus, this study aimed to (i) identify gestational age-dependent changes in the gene expression of key steroidogenic enzymes and (ii) explore the role of trophoblast cells in steroid biosynthesis and metabolism. Quantitative and Droplet Digital PCR analysis of 12 selected enzymes was carried out in the first trimester (*n* = 13) and term (*n* = 20) human placentas. Primary trophoblast cells (*n* = 5) isolated from human term placentas and choriocarcinoma-derived cell lines (BeWo, BeWo b30 clone, and JEG-3) were further screened for gene expression of enzymes involved in placental synthesis/metabolism of steroids. Finally, de novo steroid synthesis by primary human trophoblasts was evaluated, highlighting the functional activity of steroidogenic enzymes in these cells. Collectively, we provide insights into the expression patterns of steroidogenic enzymes as a function of gestational age and delineate the cellular origin of steroidogenesis in the human placenta.

## 1. Introduction

The placenta is a crucial steroidogenic organ that in the prenatal period undertakes essential functions to ensure both its own and the fetal development. The human placenta is of villous type, with the maternal–fetal interface provided by the multinucleated syncytiotrophoblast (STB) layer, a result of cell division, differentiation, and fusion of villous cytotrophoblasts (CTBs) [1]. Bathed in maternal blood, placental villi secrete numerous hormones into the maternal bloodstream, thus influencing maternal physiology, and into the fetal circulation, ensuring proper fetal development and programming [2]. In addition, the placenta can also function as an enzymatic barrier, converting cortisol to cortisone, hence protecting the fetus against excessive cortisol concentrations from maternal circulation [3,4,5].

**Figure 1:**
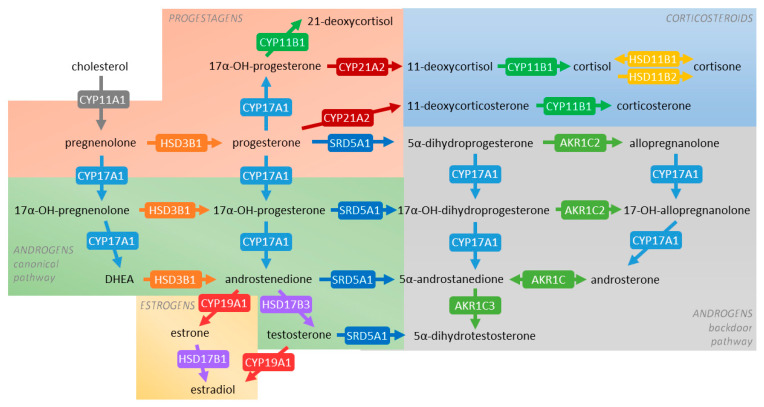
General schematic overview of steroidogenic pathways occurring in human tissues. Pathways investigated in this study include those associated with the synthesis of progestagens (orange box), corticosteroids (blue box), androgens (canonical pathway—green box, backdoor pathway—grey box), and estrogens (yellow box). The arrows are labeled with the metabolizing enzyme and, where possible, the principal reported isoform in the human placenta. Different colors are used to emphasize the involvement of individual enzymes in multiple steroidogenic pathways.

It is well accepted that during pregnancy, the placental STB layer primarily secretes progesterone and estrogens, whereas fetal organs are a source of androgens and corticosteroids [6,7] (for an overview on general steroidogenic pathways, see Figure 1). Progesterone, the key steroid implicated in the maintenance of pregnancy, exerts its functions by participating in immunotolerance [8], decidualization of the endometrium [9], and regulating trophoblast invasion [10]. Androgens are important in modulation of maternal vasculature, endothelial cell proliferation, and development of sexual characteristics [11]. Additionally, androgens serve as precursors of estrogens, which, in turn, are key in promoting embryo implantation and angiogenesis [12]. Concurrently, glucocorticoids regulate processes, such as metabolic homeostasis, inflammatory and immune reactions, and promote trophoblast proliferation and invasion [13].

Importantly, dysregulated steroid balance has been implicated in several pathologies. Exposure of the growing fetus to excess glucocorticoids represents the foundation of Barker’s theory on fetal programming, implying predisposition to a wide range of cardiovascular and metabolic diseases in adulthood [14,15]. Therefore, proper maternal–placental–fetal communication is crucial for the intercompartmental transfer of different steroid species. Moreover, as these hormones are subjected to biotransformation, the expression and activity of steroidogenic enzymes must be tightly regulated.

Steroidogenic enzymes fall into two major classes of proteins, specifically, the cytochrome P450 superfamily (CYPs) and hydroxysteroid dehydrogenases (HSDs) [16] (Figure 1). So far, the placental expression of these enzymes has been only partly described. Controversies remain regarding the expression of several rate-limiting enzymes, including CYP17A1, involved in the metabolism of pregnenolone and progesterone, and CYP21A2, involved in the metabolism of progesterone and 17α-hydroxyprogesterone [6]. Notably, physiological changes regulating the expression of steroidogenic enzymes in the placenta are poorly understood.

For a long time, the STB layer has been regarded as a functional unit accounting for placental metabolic and transport activities. The integrity and role of the CTB layer were presumed to decrease with gestation [17]. Nonetheless, latest research suggests CTBs to be the most metabolically active cells in human term placenta [18], raising the attention on the physiological relevance of CTBs to the overall human placental metabolism. Although choriocarcinoma-derived cell lines are extensively used to study placental functions, including steroid hormone synthesis and release, we, and others, have shown that these cell lines exhibit considerable differences in gene expression when compared to primary trophoblast cells isolated from human placenta [19,20,21]. Hence, choosing the right cellular model to study human trophoblast hormone synthesis and release is fundamental for a conclusive understanding of the physiological processes involved.

Thus, the current research was designed to fill the gaps in our knowledge of steroidogenesis in the human placenta and placental cell models. A comprehensive evaluation of the expression of steroidogenic enzymes in the human placenta revealed developmental regulation occurring during gestation. Comparison between primary human trophoblast cells isolated from term placenta and choriocarcinoma cell lines (BeWo, BeWo b30 clone, and JEG-3) showed dissimilar expression profiles. Additionally, a more pronounced effect of syncytialization upon induction of cyclic AMP signaling in BeWo b30 cells, compared to spontaneous fusion of CTBs in primary cells, was observed. Lastly, we revealed that primary trophoblast cells synthesize de novo a wide range of steroids, highlighting the role these cells play in the overall prenatal steroidogenesis.

## 2. Results

### 2.1. Clinical Characteristics of Mothers and Newborns Included in the Study

Thirteen first-trimester placentas after elective termination of pregnancy and 25 term placentas from healthy pregnancies without maternal use of medications were included in the study. Available clinical characteristics of the pregnancies are listed in Table 1. No statistical differences were found in the mean maternal age and pre-pregnancy BMI between the two groups, whereas gestational age was significantly different (*p* < 0.001). The first-trimester cohort has been used before to determine other aspects of placental physiology [21]. As there were no statistically significant differences between samples from smokers and nonsmokers within our first-trimester cohort, smokers were included in the study.

### 2.2. PCR Analysis of Placental Steroidogenic Enzyme Signature during Gestation

qPCR screening of 12 enzymes associated with steroid metabolism in the human placenta was initially performed. Selected enzymes included those involved in the metabolism of cholesterol (*CYP11A1*), sex steroids (*CYP17A1*, *CYP19A1*, *HSD3B1*, *HSD17B1*, *SRD5A1*, *AKR1C2*, *AKR1C3*), and corticosteroids (*CYP21A2*, *CYP11B1*, *HSD3B1*, *HSD11B1*, *HSD11B2*). Figure 1 shows an overview of the steroidogenic pathways in which these enzymes are implicated.

**Figure 2:**
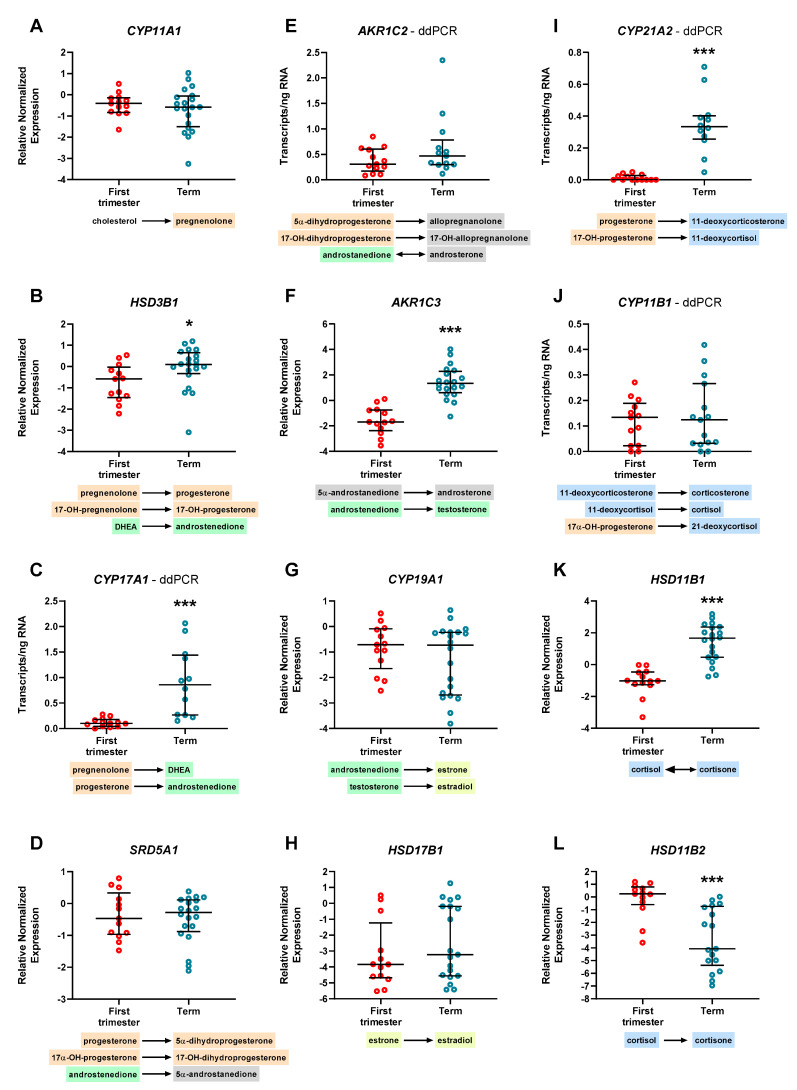
Differential gene expression of steroidogenic enzymes in the human placenta during gestation (**A**–**L**). Pathways included in the analysis are implicated in the metabolism of progestagens (orange), androgen synthesis (canonical pathway—green, backdoor pathway—grey), estrogen production (yellow), and metabolism of corticosteroids (blue). *CYP17A1* (**C**), *AKR1C2* (**E**), *CYP21A2* (**I)**, and *CYP11B1* (**J**), whose detection by qPCR analysis was not successful, were evaluated by the droplet digital PCR (ddPCR) method. The remaining genes were analyzed by qPCR technique, and relative expression was normalized to the geometric mean of TATA-box binding protein (*TBP*) and tyrosine 3-monooxygenase/tryptophan 5-monooxygenase activation protein zeta (*YWHAZ*) as reference genes. Data are shown as individual values and median with interquartile range. qPCR results are presented in logarithmic scale, whereas ddPCR data are shown in linear scale; *n* = 13 for first-trimester tissue and *n* ≥ 13 for term placenta. Statistical analysis was performed using the nonparametric Mann-Whitney test; * (*p* ≤ 0.05), *** (*p* ≤ 0.001).

Human placenta expressed *CYP11A1* (Figure 2A), *SRD5A1* (Figure 2D), *CYP19A1* (Figure 2G), and *HSD17B1* (Figure 2H) consistently throughout gestation; on the other hand, gene expression of *HSD3B1* (Figure 2B), *AKR1C3* (Figure 2F), and *HSD11B1* (Figure 2K) was significantly lower in the first-trimester placenta compared with term placenta, whereas *HSD11B2* (Figure 2L) expression was significantly downregulated at term. *CYP17A1*, *AKR1C2*, and *CYP21A1* were detected only in a fraction of samples tested, while *CYP11B1* was undetectable in all samples, using qPCR analysis.

As the gene expression profile of some genes may have been influenced by qPCR detection limits, we took advantage of the highly sensitive Droplet Digital PCR (ddPCR) methodology and quantified the absolute number of transcripts. Applying this method, we were able to demonstrate the expression of *CYP17A1* (Figure 2C), *AKR1C2* (Figure 2E), *CYP21A2* (Figure 2I), and *CYP11B1* (Figure 2J) in the human placenta. Additionally, the expression of *CYP17A1* and *CYP21A2* was significantly upregulated at term.

**Figure 3:**
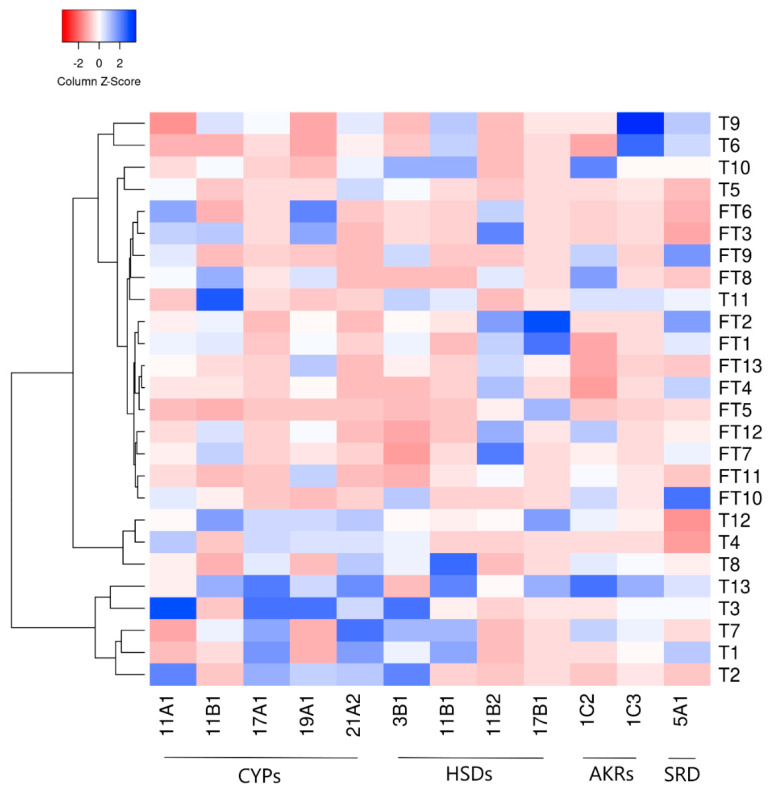
Heatmap representing the overall mRNA expression of enzymes involved in steroid metabolism in the human placenta during gestation, evaluated by quantitative and Droplet Digital PCR analysis. Enzymes are depicted along the *X* axis and comprise different classes, including members of the cytochrome P450 superfamily (CYPs), hydroxysteroid dehydrogenases (HSDs), aldo-keto reductases (AKRs), and one isoenzyme of steroid 5α-reductase (SRD). First trimester (FT) and term (T) human placenta samples are hierarchically clustered (average linkage, Euclidean distance) on the *Y* axis. Grouping of samples with similar expression levels reveals a physiological regulation of steroidogenic enzyme expression during gestation. Nonetheless, the involvement of other factors cannot be excluded. The color intensity indicates expression levels: red—downregulation, blue—upregulation.

To confirm the gestational-age-dependent regulation of steroidogenic enzyme expression in the human placenta, gene expression patterns were visualized by heatmap analysis and clustered hierarchically. Figure 3 identifies two main clusters, one thereof represented by term samples, whereas one cluster is further divided into two sub-clusters of term samples and one sub-cluster with almost exclusively first-trimester tissues. This suggests that the gestational age influences the overall gene expression of the enzymes involved in steroid metabolism. Nonetheless, the involvement of other factors contributing to these enzymes’ general expression during gestation cannot be excluded.

### 2.3. Expression Profile of Steroidogenic Enzymes in Primary Cells Isolated from Human Term Placenta and Choriocarcinoma-Derived Cell Lines

**Figure 4:**
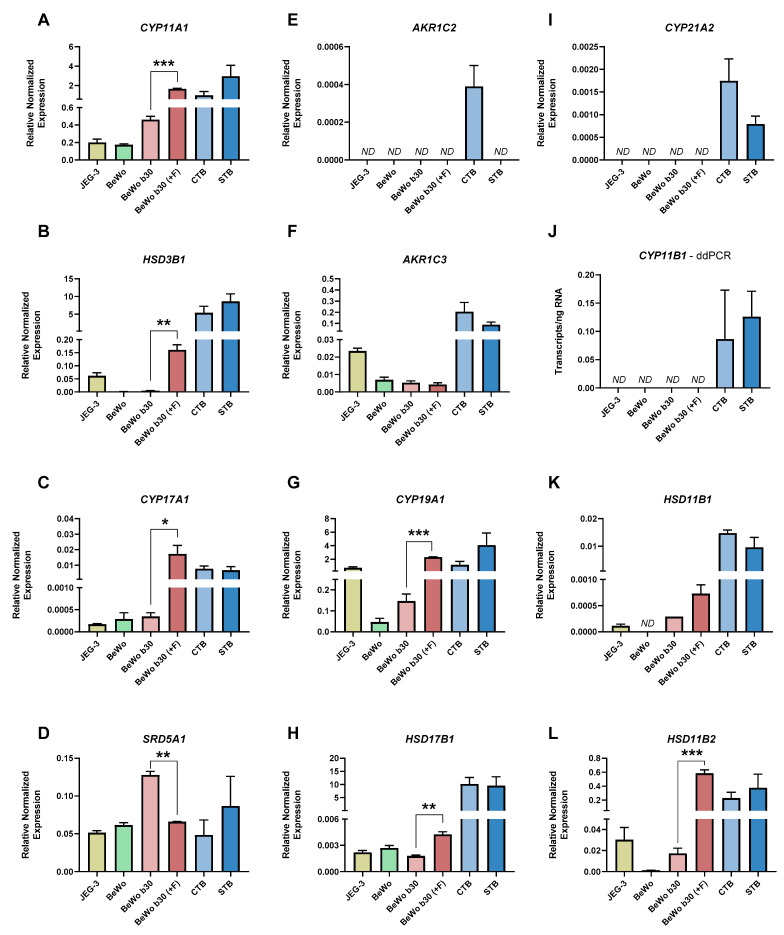
Steroid enzyme signature in choriocarcinoma-derived cell lines (BeWo, BeWo b30 clone, and JEG-3) and primary cells isolated from human term placenta (CTB/STB) (**A**–**L**). Furthermore, the effect of differentiation in BeWo b30 cells (achieved via forskolin induction; +F) and primary placental cells (CTBs spontaneously fusing in culture to form STB) is shown. *CYP11B1* (**J**), whose detection by qPCR analysis was not feasible, was evaluated by ddPCR method. The remaining genes were analyzed by qPCR technique, and relative expression was normalized to the geometric mean of TATA-box binding protein (*TBP*) and tyrosine 3-monooxygenase/tryptophan 5-monooxygenase activation protein zeta (*YWHAZ*) as reference genes. Data are shown as mean with SEM; *n* = 3 for choriocarcinoma cells and *n* = 5 for CTB/STB. Statistical analysis between differentiated and undifferentiated cells was performed using paired *t*-test; * (*p* ≤ 0.05), ** (*p* ≤ 0.01), *** (*p* ≤ 0.001).

Primary cells isolated from human term placenta (CTB and STB stage) and choriocarcinoma-derived cells BeWo, BeWo b30 clone, and JEG-3 expressed most steroidogenic enzymes tested; nonetheless, the pattern of expression differed. Apart from *SRD5A1* (Figure 4D), which showed similar expression in all cells, the rest of the enzymes tested revealed a more abundant expression in isolated primary CTB and STB. Notably, the expression of *CYP11A1* (Figure 4A), *CYP17A1* (Figure 4C), *AKR1C3* (Figure 4F), *HSD17B1* (Figure 4H), and *HSD11B2* (Figure 4L) was several-fold higher in the primary cells compared with choriocarcinoma-derived cell lines. Interestingly, while the expression of *CYP19A1* was comparably low in BeWo cells (including b30 clone), JEG-3 cells exhibited similar expression levels as CTB and STB (Figure 4G). Additionally, BeWo cells showed minimal expression of *HSD3B1* (Figure 4B), *HSD11B1* (Figure 4K), and *HSD11B2* (Figure 4L), key enzymes in the production/metabolism of active hormones. On the other hand, the expression of *AKR1C2* (Figure 4E), *CYP21A2* (Figure 4I), and *CYP11B1* (Figure 4J) was undetectable in all choriocarcinoma cells tested, with *AKR1C2* further being expressed exclusively in CTB and not in the differentiated counterpart.

Besides baseline differences in gene expression, BeWo b30 cells further showed divergent effects of differentiation on the expression of enzymes involved in steroid metabolism. Specifically, the expression of *CYP11A1* (Figure 4A), *HSD3B1* (Figure 4B), *CYP17A1* (Figure 4C), *CYP19A1* (Figure 4G), *HSD17B1* (Figure 4H), and *HSD11B2* (Figure 4L) was significantly upregulated upon induction of cyclic AMP signaling by forskolin. On the other hand, *SRD5A1* (Figure 4D) was significantly downregulated in the differentiated BeWo b30 cells. These effects were not observed when comparing CTB and STB, where the differentiation process occurs spontaneously, i.e., in the absence of forskolin stimulation (Figure 4).

### 2.4. Secretion of Steroids from Primary Trophoblast Cells in Basal State

**Figure 5:**
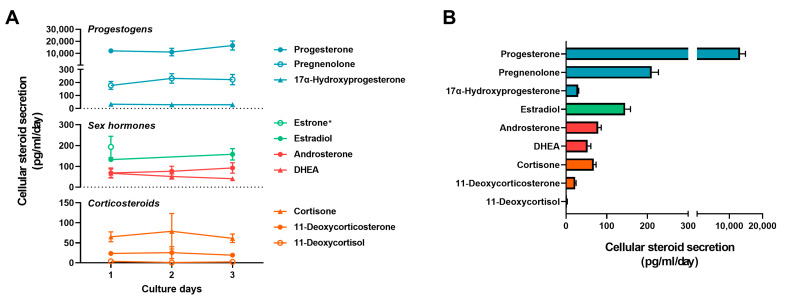
Nature of steroid synthesis and release by primary trophoblast cells. The concentration of steroids was evaluated in the cell supernatant over three days in standard culturing conditions, with daily replacement of media (**A**). Mean daily steroid release shows the predominance of progestagens as products of primary trophoblast cells, followed by several sex hormones and corticosteroids (**B**). Data are shown as mean with SEM; *n* = 10 of biological replicates with >3 values for each culture day. * Concentrations of estrone are only available for culture day one.

Intrinsic de novo synthesis of steroids was evaluated in isolated primary trophoblast cells grown on conventional plates or as a monolayer, and cultivated in standard media, i.e., without additional supplementation of precursors. Of 19 steroid species screened, 10 steroids were identified in the culture media. This secretion was stable over time (Figure 5A), indicating that even in standard culture media, with moderate cholesterol concentrations, the cells can maintain steroid homeostasis, i.e., synthesis and release (Figure 5B). We observed a high release of progestagens, with progesterone levels exceeding those of other steroid species (>10 ng/mL) and 40-fold lower pregnenolone concentrations. Interestingly, while secreted at much lower levels, we detected stable concentrations of 17α-hydroxypregnenolone released by trophoblast cells during three days of culturing. While we did not detect the downstream metabolites of 17α-hydroxypregnenolone, androstenedione, and testosterone, we observed significant release of sex hormones including dehydroepiandrosterone (DHEA), androsterone, estrone, and estradiol. Similarly, we quantified substantial release of corticosteroids in varying concentrations: cortisone > 11-deoxycorticosterone > 11-deoxycortisol. Corticosterone and cortisol were not detected.

## 3. Discussion and Conclusions

**Figure 6:**
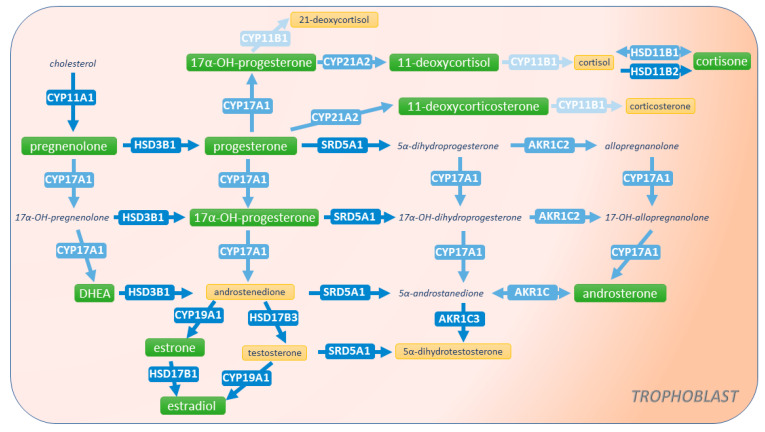
Summary of steroidogenic pathways in primary trophoblast cells isolated from human term placenta. The arrows are labeled with the metabolizing enzyme isoform tested in the study. Placental trophoblast cells express the enzymatic machinery necessary for the synthesis, metabolism, and secretion of several steroids. Nonetheless, the relative and absolute enzyme expression differs and is indicated by the blue color intensity (lighter color depicting lower expression). Identified steroid species synthesized de novo and released by trophoblast cells are highlighted in green. Metabolites whose concentrations were below the detection limits are highlighted in yellow, whereas steroids not measured in this study are depicted in italics. We also detected the gene expression of enzymes associated with the androgen backdoor pathway, but their functional activity in primary cells remains to be determined.

Balanced steroidogenesis in the maternal–placental–fetal unit plays a pivotal role in pregnancy maintenance and fetal growth and development [6]. Central to this interface, the placenta ensures proper communication between the maternal and fetal compartments [22]. In addition, it maintains steroid homeostasis by its own synthesis, metabolism, and transport of cholesterol, sex hormones, and corticosteroids. A large body of evidence sustains the importance of the placenta in the secretion of progesterone and estrogens [6,7,23]. On the other hand, the human placenta has been deemed incapable of androgen production, rendering it dependent on maternal and fetal sources [6]. Only recent investigations of the backdoor steroid pathways of the fetoplacental unit hinted that the placenta itself participates in androgen metabolism [11]. In this study, we bring new insights into placental steroidogenesis by characterizing in detail the expression of enzymes involved in the synthesis and metabolism of sex hormones and corticosteroids. Further, we focused on the placental cell models (BeWo, BeWo b30 clone, JEG-3, and primary human trophoblast cells isolated from term placenta) currently used to study placental steroidogenic functions. We found that primary trophoblast cells are the most adequate model expressing the necessary machinery for steroid synthesis, metabolism, and secretion (summarized in Figure 6).

Controversy exists in the literature regarding placental CYP17A1 expression and functional activity, a key enzyme metabolizing pregnenolone or progesterone to produce DHEA or androstenedione, respectively [16]. Several studies have deemed the placenta as incapable of de novo androgen synthesis due to a supposed lack of CYP17A1 expression and activity [24,25]. Thus, the current dogma suggests that placental production of estrogens relies on fetal and, to a lesser extent, maternal androgen precursors, specifically DHEA-sulfate. Despite this, research dating from 1961 [26] as well as a more recent work [27] have documented functional expression and activity of CYP17A1 in human placental homogenates or trophoblast cells, respectively. This has suggested placental CYP17A1 activity as an important source of androgen precursors within the tissue. In line with these studies, we detected with qPCR analysis considerable expression of *CYP17A1* in isolated primary trophoblasts and the choriocarcinoma-derived cell lines JEG-3 and BeWo. Moreover, using ddPCR analysis, we reported, for the first time, *CYP17A1* transcripts in human placenta, with minimal expression in the first-trimester samples and upregulation at term. This finding fully reflects the knowledge from rodent models, where CYP17 expression increases slowly and progressively during gestation, reaching the peak at gestation day 18 and declining just before parturition [28]. Likewise, it has been reported that human plasma concentrations of 17α-hydroxyprogesterone, a CYP17A1 product, significantly increase at term [29]. Lastly, it is known that, at the beginning of pregnancy, estrogen secretion is maintained by the corpus luteum, with the placenta almost exclusively taking up the role thereafter [23]. Considering the placenta mass, blood flow, and steroid precursors, Escobar et al. suggested that 20–30% of the estrogen synthesis to be derived from placental CYP17A1 function and androgen generation [27]. Thus, *CYP17A1* upregulation at term may reflect the increasing placental contribution in the generation of androgen precursors, and favoring CYP19A1-mediated estrogen synthesis, important for fetal sex development [30].

In addition to *CYP17A1* expression, we demonstrated a release of 17α-hydroxyprogesterone by primary trophoblast cells cultured in standard media. A similar finding has been reported earlier [27], suggesting CYP17A1 functional activity. This statement is further supported by the fact that a high 17α-hydroxyprogesterone concentration was found in the retroplacental space and significantly higher levels in the fetal umbilical vein compared with the umbilical artery [29,31]. Interestingly, however, in the same culturing method, we observed a high release of other progestagens and androgens; specifically, progesterone > pregnenolone > androsterone > DHEA, whereas levels of androstenedione were below the detection limit. The high concentration of progesterone released by trophoblast cells was expected since HSD3B1, which is vastly expressed in the placenta, shows at the same time high substrate affinity for pregnenolone (*K*_m_ = 0.24 µM). Altogether, this favors the oxidative reaction [32]. Importantly, we observed upregulation of *HSD3B1* expression at term, which corresponds to data by others [23], reporting increased progesterone blood levels throughout pregnancy, with the highest levels observed during the last four weeks of gestation. Nonetheless, androgen species, released by trophoblast cells and detected in our system, contrasted with the findings reported by Escobar et al. [27]. There, it was suggested that, in the human placenta, the Δ4 pathway of androgen synthesis (leading to androstenedione) is preferred to the Δ5 route (generating DHEA). Nevertheless, our data on DHEA production by primary cells agree with the reported human CYP17A1 enzyme kinetics, specifically the preference for 17α-hydroxypregnenolone as a substrate for yielding DHEA [16]. Moreover, apart from the classical route of 5α-dihydrotestosterone synthesis from testosterone, a backdoor pathway was recently characterized. The latter was shown to be essential for male sexual development [33]. Interestingly, androsterone was reported as the main backdoor androgen, but not originating from gonadal tissues. In contrast, synthesis by nongonadal tissues (including placenta) was speculated [11].

Apart from CYP17A1, the key enzymes associated with entry on the backdoor pathway are the steroid 5α-reductase type 1 (SRD5A1) and the aldo-keto reductase 1C2 (AKR1C2). A previous study suggested that, relative to tissue weight, the human placenta expresses SRD5A1 around 1000 times more than the testis [11]. We observed both enzymes’ expression in the human placenta and trophoblast cells, with *AKR1C2* expression being detected only in the CTB state. These two enzymes are undoubtedly functionally active in the placenta since high levels of backdoor metabolites, especially 5α-dihydroprogesterone and allopregnanolone, are reported in the human placenta [11]. Although limited by technical restraints of measuring backdoor metabolite levels in cell supernatants, but based on lack of androstenedione release, we hypothesize that the backdoor pathway is functionally active and may contribute to the observed androsterone release by primary trophoblast cells. Further research is required to fully demonstrate the pathways involved in placental androsterone synthesis.

Notably, we showed that primary trophoblast cells are capable of de novo estrone and estradiol synthesis when maintained in standard culture media. Although androstenedione and testosterone concentrations in our system were below detection limits, we suggest that estrogen synthesis in primary cells occurs chiefly via utilization of androstenedione, the preferred substrate of CYP19A1 [34]. Our findings, and those by Escobar et al. [27], in an in vitro system devoid of maternal and fetal contribution, demonstrate exclusive estrogen synthesis by placental trophoblast cells. Such a phenomenon highlights the placental ability to maintain estrogen homeostasis even when maternal and fetal androgen precursors are absent. Collectively, as also suggested before [11,27], we postulate that placental biosynthesis of androgens is an essential source of placental estrogen precursors and androsterone for fetal masculinization.

Recent studies have shed light on novel aspects concerning the importance of steroid hormone homeostasis and interplay during pregnancy. In particular, it has been shown that both progesterone and glucocorticoids act on glucocorticoid receptors in T cells, triggering immunoregulatory signals [35]. Steroid hormone bioavailability is believed to be the major factor determining the equilibrium between progesterone and glucocorticoids, which, in turn, is tightly regulated by the expression of steroidogenic enzymes [36]. While in humans, total cortisol levels increase until mid-gestation and then remain stable until delivery, availability, and free cortisol concentration continue to rise throughout gestation [36]. Lipophilic in nature, cortisol is believed to freely cross the placenta, while fetal protection is maintained by the activity of placental HSD11B2 [5]. Nonetheless, we observed placental *HSD11B2* expression, mediating deactivation of cortisol to cortisone, to significantly decrease at term. On the other hand, expression of *HSD11B1*, functioning in both directions (preferably reduction of cortisone to cortisol [16]), is upregulated at term. This feature of HSD11Bs was also previously reported, both in the placenta [3] and fetal membranes [37], and was considered as a mechanism involved in the initiation of parturition [38].

Notwithstanding the documented expression, the functional involvement of placental HSD11B1 in cortisol homeostasis is puzzling. Using dual human placenta perfusion, de novo cortisol synthesis has recently been suggested and attributed to HSD11B1 [4]. Contrary to Sun et al. [39], who found *HSD11B1* expression in the extravillous trophoblasts and endothelial cells, we detected *HSD11B1* mRNA expression in both CTB and STB. Nonetheless, while we detected a high release of cortisone by primary trophoblast cells, cortisol levels were below the detection limits. This corresponds to the expression data in trophoblast cells, where several-fold higher transcripts of *HSD11B2* were recorded. While it is intriguing to assume that the cortisol-regenerating function of HSD11B1 may provide a fine-tuned mechanism controlling cortisol levels in the fetoplacental unit [37], the lower expression and substrate affinity of this enzyme compared to HSD11B2 and the lack of cortisol release by primary trophoblast cells, challenge this concept. Additionally, the high placental activity of NADPH-dependent CYPs drives the oxidation of NADPH and, thus, increases NADP^+^ availability [40]. Albeit with lower affinity, this may promote the oxidative activity of HSD11B1-inactivating glucocorticoids.

Interestingly, we observed the release of 11-deoxycorticosterone and 11-deoxycortisol by primary trophoblast cells. These glucocorticoids are generated from progesterone and 17α-hydroxyprogesterone, respectively, via the activity of CYP21A2. Subsequently, they are subject to metabolism by CYP11B1, forming corticosterone and cortisol [16]. To date, both enzymes have been deemed as originating from adrenals [16]. Interestingly, herein, we report for the first time *CYP21A2* and *CYP11B1* expression in human placenta and primary trophoblast cells. While CYP11B1 was expressed in low levels throughout gestation, CYP21A2 showed gestational age-dependent changes with minimal expression in the first trimester and significant upregulation at term. Based on expression patterns and intrinsic de novo steroid release in vitro, we confine functional CYP21A2 in the trophoblast cells. We suggest that, under physiological conditions, CYP11B1 is functionally inactive in these cells.

Collectively, we reported and discussed novel aspects of steroidogenesis in the human placenta and physiological regulation occurring during gestation. Importantly, we provide evidence on the contribution of placental trophoblast cells in the maintenance of balanced steroid homeostasis during perinatal development. Altered levels of steroid hormones have been associated with pregnancy complications, including preeclampsia and gestational diabetes [6]. Placental dysfunction leading to intrauterine growth restriction also appears to affect the sexual development of male external genitalia [41]. In contrast, overexposure of the female fetus to androgens is considered a risk factor for developing inappropriate phenotypes at birth, such as virilized genitalia [42]. Additionally, evidence exists that certain HSD11B1 and CYP19A1 gene polymorphisms are associated with hypertensive pregnancy disorders [43,44]. Thus, understanding the complexity of physiological dynamics in steroid biosynthesis, metabolism, and release is fundamental to delineate the biological importance of steroids in maintaining pregnancy.

## 4. Materials and Methods

### 4.1. Human Placenta Collection

Human placenta samples were collected at the Department of Obstetrics and Gynecology, University Hospital in Hradec Kralove, Czech Republic, or at the Division of Gynecology and Obstetrics, Lindenhofgruppe, Bern, Switzerland. First-trimester placentas (*n* = 13; 8–11 weeks gestation) were obtained after elective interruption of a healthy pregnancy, whereas term placentas (*n* = 25; 38–40 weeks gestation) from uncomplicated pregnancies immediately after delivery. All experiments were performed following the Declaration of Helsinki and were approved by the University Hospital Research Ethics Committee (201706 S17P; 06/06/17) and Ethics Committee of the Canton of Bern (Basec Nr. 2016-00250; 14/04/2016). Informed consent was obtained from all subjects. Clinical characteristics of pregnancies included in the study are shown in Table 1.

### 4.2. Primary Cell Isolation and Analysis of Cell Purity

Primary trophoblast cells were isolated from term placental tissue (*n* = 10), as previously described [20,21]. Briefly, villous tissue was subjected to three steps of 30 min enzymatic digestion with 0.25% trypsin (Sigma-Aldrich, St. Louis, MO, USA) and 300 IU/mL deoxyribonuclease I (Sigma-Aldrich, St. Louis, MO, USA) at 37 °C. The cell suspension was collected in Dulbecco’s Modified Eagle Medium (high glucose) and overlaid on a discontinuous Percoll^®^ gradient (Sigma-Aldrich, St. Louis, MO, USA). Collected CTB cells were cultured in Dulbecco’s Modified Eagle Medium (high glucose) supplemented with 10% FBS and 1% antibiotic–antimitotic (Thermo Fisher Scientific, Waltham, MA, USA). As isolated CTBs spontaneously fuse in culture to form the STB, culturing 12 h represents the CTB stage [45]. In contrast, the STB stage was collected at 72 h after isolation, with daily change of medium.

The purity of the isolated CTBs was evaluated by flow cytometry as previously described [21]. Dual staining with directly-labeled antibodies (Novus Biologicals, CO, USA) was carried out for the following proteins: AF 488^®^ anti-cytokeratin 7, AF 647^®^ anti-vimentin, and AF 488^®^ anti-E-cadherin. At least 10,000 cells were acquired by flow cytometry (BD FACS LSRII; BD Biosciences, San Jose, CA, USA). Data analysis was performed using BD FACSDiva^™^ (BD Biosciences, San Jose, CA, USA) and FlowJo^®^ software version-10 (FlowJo LLC, Ashland, OR, USA). The cells stained with positive epithelial cell markers, cytokeratin 7 [46] and E-cadherin [47], comprised 86.1 ± 9.5% and 79.5 ± 5.6%, respectively. Staining with vimentin for mesenchymal cells, fibroblast, and stromal cells [47] showed 18.9 ± 8.7% of the whole isolated cellular population.

### 4.3. Cells

Choriocarcinoma-derived BeWo and JEG-3 cell lines were obtained from the European Cell Culture Collection (Salisbury, Wiltshire, UK). BeWo cells were cultured at 37 °C/5% CO_2_ in Ham’s F-12 medium supplemented with 10% FBS, whereas JEG-3 were cultured in Dulbecco’s Modified Eagle Medium (high glucose) supplemented with 10% FBS. BeWo b30 cell line was obtained from Dr. A. Schwartz (Washington University, St. Louis, MO, USA) and were cultured in the same medium as JEG-3 cells. BeWo b30 cell line was further treated with 100 µM forskolin for 72 h to induce differentiation [21]. In this case, the medium was changed daily.

### 4.4. RNA Isolation, Reverse Transcription

According to the manufacturer’s instructions, RNA isolation was performed from human placental tissue and cells using Tri Reagent (Molecular Research Centre, Cincinnati, OH, USA) or Trizol (Invitrogen, Carlsbad, CA, USA). Absorbance (A) was measured using NanoDrop™ 1000 Spectrophotometer (Thermo Fisher Scientific, Waltham, MA, USA). Absorbance at 260 nM was used to calculate the RNA concentration. On the other hand, A260/280 and A260/230 ratios were used to evaluate RNA purity and potential contamination by DNA, protein, and organic solvents. Reverse transcription was performed in 1 µg of isolated RNA, in a total volume of 20 µL, using the iScript cDNA Synthesis Kit (Bio-Rad, Hercules, CA, USA) or GoScript™ Reverse Transcriptase System (Promega, Madison, WI, USA) according to the manufacturer’s instructions.

### 4.5. PCR Analysis

#### 4.5.1. Quantitative PCR Analysis

qPCR analysis was performed in QuantStudio™ 6 (Thermo Fisher Scientific, Waltham, MA, USA). Thermal conditions were set according to the manufacturer’s instructions for the TaqMan^®^ Universal Master Mix II without UNG (Thermo Fisher Scientific, Waltham, MA, USA) and predesigned TaqMan^®^ assays (listed in Table S1). A total of 10 ng/µL of cDNA was amplified in triplicate in a 5 µL volume (384-well plate). RefFinder (https://www.heartcure.com.au/reffinder (accessed on 7 December 2020)) [48] was used to identify reference genes for which the expression was invariant between the first-trimester and term tissues. After evaluation, target gene expression was normalized against the reference genes TATA-box binding protein (*TBP*; Thermo Fisher Scientific, Waltham, MA, USA) and tyrosine 3-monooxygenase/tryptophan 5-monooxygenase activation protein zeta (*YWHAZ*; Thermo Fisher Scientific, Waltham, MA, USA) using the ΔΔCt method [49].

#### 4.5.2. Droplet Digital PCR Analysis

Absolute quantification of undetectable genes in qPCR analysis was performed using duplex ddPCR analysis, as described previously [50]. Briefly, the reaction mixture consisted of 10 µL of ddPCR™ Supermix for Probes (Bio-Rad, Hercules, CA, USA) and 1 µL of each of the predesigned probe assays (listed in Table S1); 1 µL of cDNA (concentration 50 ng/µL) was used in a total volume of 20 µL. Droplets were generated using the QX200 Droplet Generator (Bio-Rad, Hercules, CA, USA) and amplified to end-point using a T100™ Thermal Cycler (Bio-Rad, Hercules, CA, USA). The thermal conditions used were set by the manufacturer. Droplet counting was performed in the QX200™ Droplet Reader (Bio-Rad, Hercules, CA, USA), and the concentration of the target gene was calculated using the QuantaSoft™ Software (Bio-Rad, Hercules, CA, USA).

### 4.6. Extraction of Steroids from Cell Culture Supernatants

Isolated CTBs were cultured in Dulbecco’s modified Eagle medium (high glucose) supplemented with 10% FBS and 1% antibiotic–antimitotic (Thermo Fisher Scientific, Waltham, MA, USA). Cells were seeded at a density of 0.2 × 10^6^ cells/cm^2^ in CellBIND 6-well plates (Falcon^®^, Corning, NY, USA), or at a density of 1 × 10^6^ cells/cm^2^ in 12-well Transwell^®^ inserts with 0.4 μm pore PET membranes (Falcon^®^, Corning, NY, USA) coated with Matrigel^®^ (BD Biosciences, San Jose, CA, USA) at a concentration of 25 μg/cm^2^ before the experiment. Two steroid extraction procedures were applied, depending on the method used to analyze steroids.

For column extraction, the cell culture supernatants were replaced daily and collected on days 1, 2, and 3. A total of 10 mL of cell culture supernatants were first concentrated by passing through Sep Pak 18 columns (Waters, Hertfordshire, UK). After the extraction, columns were dried with nitrogen gas, and steroids were eluted with 5 mL methanol. Then the samples were processed by using AbsoluteIDQ^®^ stero17 kit as described below.

For liquid–liquid extraction, methanol:dicholoromethane (1:2; Sigma-Aldrich, St. Louis, MO, USA) was added to the cell supernatants and mixed on wheel for 20 min. For the liquid phase separation, all tubes were centrifuged at 1560× *g* for 10 min at 4 °C. The organic phase was collected in a fresh tube and evaporated for 5 min at 56 °C with nitrogen gas. After drying, the samples were reconstituted in 200 µL of 30% methanol. Finally, steroid metabolites were analyzed by LC–MS/MS using an in-house method as described below.

### 4.7. Measurement of Steroids in Cell Supernatant

The following steroids were measured in this study, namely: cortisol, cortisone, 11-deoxycortisol, 21-deoxycortisol, aldosterone, corticosterone, 11-deoxycorticosterone, 17α-hydroxyprogesterone, progesterone, pregnenolone, estradiol, estrone, androstenedione, androsterone, DHEA, dehydroepiandrosterone sulfate, dihydrotestosterone, etiocholanolone, and testosterone. Steroid quantification was performed either using the AbsoluteIDQ stero17 kit (Biocrates, Innsbruck, Austria) or an in-house developed method. For the AbsoluteIDQ stero17 kit, samples were prepared according to the instructions of the manufacturer analyzed by LC–MS/MS using a Waters 2D Acquity I-Class UPLC coupled to a Waters Xevo TQ-S mass spectrometer (Waters, Hertfordshire, UK). Data analysis was performed by using TargetLynx (Waters, Hertfordshire, UK). The in-house method was based on the approach developed by Peitzsch et al. [51]. Briefly, samples were spiked with isotopically labeled standards, followed by addition of 250 µL of zinc sulphate (0.1 mol/L) and 500 µL of cold methanol (−20 °C) for protein precipitation and steroid extraction. The samples were vortexed and centrifuged at 8000× *g* for 5 min. A total of 250 µL of water was added to each sample and purified by solid phase extraction on an OasisPrime HLB 96-Well Plate using a positive pressure 96-well processor (both Waters, Hertfordshire, UK). LC–MS measurements were performed using Vanquish UHPLC coupled to a QExactive Orbitrap Plus (both Thermo Fisher Scientific, Waltham, MA, USA). All data were processed using TraceFinder 4.0 (Thermo Fisher Scientific, Waltham, MA, USA).

### 4.8. Statistical Analysis and Graphical Presentation

The heatmap analysis with hierarchical clustering was performed using Heatmapper (http://www.heatmapper.ca/ (accessed on 7 December 2020)) [52]. All other statistical analyses and graphical presentations were implemented in GraphPad Prism 8.3.1 software (GraphPad Software, Inc., San Diego, CA, USA). Gene expression data on human placenta tissue were assessed using Mann-Whitney tests. The effect of differentiation in primary and BeWo b30 cells was evaluated using paired *t*-tests.

## Figures and Tables

**Table 1:** Clinical characteristics of pregnancies included in the study. Parameters are expressed as mean ± SD. Statistical analysis was performed using the nonparametric Mann-Whitney test; *** (*p* ≤ 0.001).

Parameter	First Trimester	Term
Number of individuals	13	25
Maternal age (years)	27.91 ± 8.04	33.16 ± 4.92
Gestational age (weeks)	9.62 ± 1.19	39.44 ± 0.91 ***
Smoking (%)	38	0
Pre-pregnancy BMI (kg m^−2^)	24.48 ± 3.31	24.18 ± 4.27
Delivery BMI (kg m^−2^)	–	28.72 ± 4.57
Labor (% caesarian section)	–	52
Birth weight (kg)	–	3.32 ± 0.36
Birth height (cm)	–	50.10 ± 1.62
Fetal sex (% male)	–	48

## Data Availability

Not applicable.

## Supplementary Materials

The following are available online at https://www.mdpi.com/1422-0067/22/4/1704/s1.

## Abbreviations

AKR	aldo-keto reductase
CTB	cytotrophoblast
CYP	cytochrome P450
DHEA	dehydroepiandrosterone
HSD	hydroxysteroid dehydrogenase
SPE	solid phase extraction
SRD	steroid 5α-reductase
STB	syncytiotrophoblast
