# Supplementary material for: Revisiting Steroidogenic Pathways in the Human Placenta and Primary Human Trophoblast Cells
Source: Int J Mol Sci. 2021 Feb 8;22(4):1704. doi: 10.3390/ijms22041704 (PMC7915605; doi:10.3390/ijms22041704)
Supplement: Supplementary file 1 [file ijms-22-01704-s001.pdf]

Revisiting steroidogenic pathways in the human placenta and primary human trophoblast cells

SUPPLEMENTARY INFORMATION

Table S1: List of predesigned probe assays used in qPCR and ddPCR analysis. All primers were obtained from Thermo Fisher Scientific, Waltham, MA, USA, except the HEX assay, which was obtained from BioRad, Hercules, CA, USA.

Gene symbol	Gene aliases	Assay ID
CYP11A1	Cholesterol Desmolase, P450SCC	Hs00167984_m1
CYP11B1	Steroid 11-Beta-Hydroxylase, Cytochrome P450C11	Hs01596406_gH
CYP17A1	Steroid 17-Alpha-Monooxygenase, Cytochrome P450c17	Hs01124136_m1
CYP19A1	Aromatase, P-450AROM, Estrogen Synthase	Hs00903411_m1
CYP21A2	Steroid 21-Monooxygenase, Steroid 21-Hydroxylase, 21-OHase	Hs00416901_g1
HSD3B1	Delta-5-3-Ketosteroid Isomerase	Hs04194787_g1
HSD11B1	Hydroxysteroid 11-Beta Dehydrogenase 1	Hs01547870_m1
HSD11B2	Hydroxysteroid 11-Beta Dehydrogenase 2	Hs00388669_m1
HSD17B1	Hydroxysteroid 17-Beta Dehydrogenase 1	Hs00166219_g1
AKR1C2	Aldo-Keto Reductase Family 1 Member C2	Hs04194036_gH
AKR1C3	Aldo-Keto Reductase Family 1 Member C3	Hs00366267_m
SRD5A1	Steroid 5 Alpha-Reductase 1	Hs00971645_g1
**Reference genes**		
TBP	TATA-box binding protein	Hs00427620_m1
YWHAZ	tyrosine 3-monooxygenase/tryptophan 5-monooxygenase activation protein zeta	Hs01122445_g1
B2M	beta-2-microglobulin	dHsaCPE5053101 (HEX)
